# Higher Perceived Stress as an Independent Predictor for Lower Use of Emotion-Focused Coping Strategies in Hypertensive Individuals

**DOI:** 10.3389/fpsyg.2022.872852

**Published:** 2022-05-24

**Authors:** Laura Aló Torres, Regina Silva Paradela, Luiza Menoni Martino, Danielle Irigoyen da Costa, Maria Claudia Irigoyen

**Affiliations:** ^1^Instituto do Coracao (InCor), Hospital das Clinicas (HCFMUSP), Faculdade de Medicina, Universidade de São Paulo, São Paulo, Brazil; ^2^Brain Institute (InsCer), Pontifícia Universidade Católica do Rio Grande do Sul (PUCRS), Porto Alegre, Brazil

**Keywords:** hypertension, psychological stress, executive function, psychological adaptation, quality of life, emotional regulation

## Abstract

**Introduction:**

Individuals with high scores of perceived stress (PS) are more likely to develop arterial hypertension (AH) than those with low levels of stress. In addition to this, AH and stress are both independent risk factors for executive function (EF) impairment and worse quality of life (QoL). Therefore, strategies to control and cope with emotional stress are of paramount importance. However, less is known about the association of PS with EF, QoL, and coping in individuals with hypertension. This study aimed to evaluate the association of PS with EF performance, coping strategies use, and QoL in a sample of hypertensive patients.

**Methods:**

We assessed a group of 45 hypertensive individuals (mean age = 58.42 ± 8.9 years, 71.11% female). The EF evaluation was: Frontal Assessment Battery; Controlled Oral Word Association Test—FAS; Letter-Number Sequencing subtest from the Wechsler Adult Intelligence Scale—Third Edition (WAIS-III); Digit Span subtest from the Wechsler Memory Scale-Revised (WMS-R) and Wisconsin Card Sorting Test. The type and frequency of coping strategies used were measured by the Brief Coping with Experienced Problems Scale (Brief-COPE). The World Health Organization Quality of Life Questionnaire Bref (WHOQOL-bref) was applied to measure QoL. The associations of the PS with EF performance, coping strategies, and QoL were investigated using univariate and multiple linear regression models adjusted for age, sex, education, systolic pressure, and depression symptoms.

**Results:**

In the multivariate analyses, higher PS was an independent predictor for a lower frequency of emotion-focused strategy use (β = −0.23; *p* = 0.03). However, PS was not significantly related to EF and Qol in this sample. The lower the PS, the greater the use of emotion-focused coping.

**Conclusion:**

Hypertensive individuals with high PS use less frequently positive emotion-focused coping strategies.

## Introduction

Individuals with high scores of perceived stress (PS) are more likely to develop arterial hypertension (AH) than those with low levels of stress (Hamer and Steptoe, 2012; Lu et al., 2019). Furthermore, hypertensive patients generally report feeling more stressed than individuals without the diagnosis of AH (Footman et al., 2013; Liu et al., 2017).

In addition to being associated with stress (Höfelmann et al., 2018; Khayyat et al., 2019; Samiei Siboni et al., 2019), AH can impact cognition in multiple domains (Gifford et al., 2013; Muela et al., 2017). There is evidence that hypertensive individuals perform worse in tests that assess executive functions (EF) compared to healthy individuals (Moraes et al., 2020, 2019). Souza-Talarico et al. (2020) also found that work-related stress was associated with lower performance on delayed recall, verbal fluency, and EF tests in middle-aged adults (Souza-Talarico et al., 2020). Therefore, AH and stress can be both independent risk factors for cognitive impairment.

Arterial hypertension and stress can also impact the quality of life (QoL) (Ames et al., 2001; Carvalho et al., 2013). Individuals with hypertension seem to have a worse QoL compared to a non-hypertensive group (Carvalho et al., 2013; Cao et al., 2018). In the same way, evidence showed that major or minor stressors events have a significant impact on the QoL of low-income patients with hypertension (Ames et al., 2001). In another sample, composed of 348 dental faculty members from master’s and doctoral programs in Brazil, Meira et al. (2020) found a negative correlation between PS and all QoL domains: physical, psychological, social, and environmental (Meira et al., 2020). However, data about the relationship between PS and QoL in hypertensive individuals is still scarce.

Because of all these deleterious effects of stress on cognition and QoL, strategies to control and deal with emotional stress are of paramount importance. The management of emotional stress can be combined with other pharmacological and non-pharmacological interventions to achieve better results, especially in individuals with AH (Issa et al., 2020).

Studies with other populations indicate that the performance of EF may be associated with the way the subject deals with stress. Lower EF performance in stroke subjects predicted more frequent use of avoidant coping (Kegel et al., 2014). In subjects who suffered from a traumatic brain injury, lower EF performance correlated with greater use of emotion-focused coping (Krpan et al., 2007). A better EF performance, in turn, was related to greater use of problem-focused coping (Krpan et al., 2007). Concerning hypertensive individuals, the instrumental dimension of the coping questionnaire, which refers to being active and task-oriented, such as searching for medical information or taking the prescribed drugs at the right moment, was positively associated with QoL (Rueda and Pérez-García, 2013). However, less is known about how the level of PS can impact the choices of coping strategies, especially in hypertensive patients.

Considering that emotional stress, besides hypertension, can impact a huge number of functions important for daily life, this study aimed to examine the association between PS and EF performance, coping strategies, and QoL in a group of hypertensive individuals. We hypothesize that PS is negatively associated with EF performance, positively associated with dysfunctional coping, negatively associated with the problem and emotion-focused coping strategies, and negatively associated with QoL domains.

## Methods

### Participants

This cross-sectional study included patients with a previous diagnosis of AH according to the 7^a^ Brazilian Arterial Hypertension Guideline (systolic BP ≥ 140 and/or diastolic BP ≥ 90) from the Hypertension Unit of the São Paulo University Heart Institute, as previously described (Paradela et al., 2021).

Of 102 participants screened with a cognitive evaluation, and a clinical and sociodemographic questionnaire (Paradela et al., 2021), 45 accomplished the eligibility criteria for this study. The inclusion criteria were age between 40 to 70 years old and previous diagnosis of hypertension. The exclusion criteria were less than 4 years of education, severe cognitive or communication impairment, estimated intelligence quotient (IQ) of less than 80, self-reported previous diagnosis of stroke, head trauma, epilepsy, and/or history of substance abuse, and incomplete data of PS questionnaire.

The estimated IQ was calculated based on the scores of the subtests Vocabulary and Cubes from the Wechsler Adult Intelligence Scale—III, a version validated for the Brazilian population (Nascimento and Figueiredo, 2002). The local ethics committee approved the protocol (local register number: 4266/15/093, 3.008.526), and all participants gave written informed consent.

### The Perceived Stress Evaluation

PS of the participants was measured using the 14-item version of the Perceived Stress Scale (PSS-14). The scale analyzes how unpredictable, unmanageable, and overwhelmed life has been in the last month through 14 questions that comprise the questionnaire (Palagini et al., 2016; Lu et al., 2019). The PSS-14 and the shorter version (PSS-10) have proved to be clear and reliable tools to evaluate the PS of the Brazilian population (Luft et al., 2007; Siqueira Reis et al., 2010). They presented suitable psychometric performance and adequate reliability and validity, supporting their use in this population (Luft et al., 2007; Siqueira Reis et al., 2010). The PSS-14 also presents a good internal consistency (*r* = 0.82). Each question is rated on a 5-point Likert scale from 0 (never) to 4 (always). The total score can range from 0 to 56. The higher the score, the greater the PS (Lu et al., 2019).

### Executive Function Evaluation

EF was evaluated using the following tests: the Frontal Assessment Battery (FAB), the FAS letters fluency from Controlled Oral Word Association Test (COWAT), Letter-Number Sequencing from the Wechsler Adult Intelligence Scale—Third Edition (WAIS-III), Digit Span Forward and Backward subtests from the Wechsler Memory Scale—R, and the Wisconsin Card Sorting Test—WCST. A detailed description of these tests can be found here (Paradela et al., 2021).

### Wisconsin Card Sorting Test

The Wisconsin Card Sorting Test (WCST) is a classical test that estimates especially the capacity for planning, the cognitive flexibility, the capacity to change in order with the presented stimulus, and the control of impulsivity. It composes of four key cards with different geometric figures drawn: one red triangle, two green stars, three yellow crosses, and four blue circles. The examiner presented the key cards to the individual. With them, the patient should match the other cards given to him. He should try to match each card for color, number, or form, depending on the current criterion not revealed to the subject. There was one rule of combination during each phase the participant did not know. After each try of the participant, the evaluator should give feedback about if the combination was correct or not (Fagundo et al., 2015). The total number of categories that each patient correctly combined was used to measure the cognitive flexibility.

To make more standardized comparisons of the cognitive tests, a Z score was calculated for each test. The participant’s test score was subtracted from the mean sample score. The difference was divided by the sample standard deviation (SD). Thus, a Z score of—1 represents a cognitive performance that is 1 SD below the mean sample score for each test (Rawlings et al., 2014; Souza-Talarico et al., 2020). A composite EF Z score was calculated by averaging the Z scores of the FAB, FAS letters fluency test, Letter-Number Sequencing, Digit span forward and backward, and completed categories from WCST and then standardizing this mean (Rawlings et al., 2014; Paradela et al., 2021).

### Coping

The 28-item Brief Coping Orientation for Problem Experienced (Brief COPE) questionnaire is a self-report questionnaire with fourteen subscales describing different coping strategies, with two items per subscale. This version is a brief form of a previously published measure called the COPE Inventory (Carver et al., 1989). The Brief COPE was developed to evaluate more quickly and easily the type and frequency of coping strategies that are used by someone (Carver, 1997; Chew et al., 2020). The Portuguese version has already been validated for the Brazilian population (Brasileiro, 2012). The test-retest rates were mostly above 0.75, demonstrating good reliability. Cronbach’s alpha for the whole Brief COPE was 0.84, proving to be a reliable instrument to assess coping strategies for use in Brazil (Brasileiro, 2012). It is composed of 28 questions and each one is rated on a 4-point Likert scale ranging from 1 (never) to 4 (very often). The questions can be grouped into three main coping responses: problem-focused (active coping, planning, and instrumental support), emotion-focused (emotional support, positive reframing, humor, religion, and acceptance), and dysfunctional coping (self-distraction, denial, substance use, behavioral disconnection, venting of emotions and self-blaming) (Cooper et al., 2006). The score of each main coping response was determined by the sum of the subscales scores. A higher score indicates more frequent use of a specific coping response (Altunan et al., 2021).

### Quality of Life

QoL was evaluated using the World Health Organization Quality of Life Questionnaire (WHOQOL-bref). This questionnaire is a short version of WHOQOL-100 (Skevington et al., 2004). The Portuguese version of the abbreviated instrument showed a good performance concerning internal consistency, discriminant validity, criterion validity, concurrent validity, and test-retest reliability in a study of application to the Brazilian population (Fleck et al., 2000). WHOQOL-bref has 26 questions and is composed of four domains (physical, psychological, social relations, and environment). The questionnaire has one first question about the overall perception of QoL and 24 other questions related to these four domains. Each question has answer options of 1 to 5. The alternatives are on a kind of Likert scale in the aspects: of intensity, capacity, frequency, and evaluation (Marcacine et al., 2019). The patient was oriented to read each question and circle the number that represented the best answer (Skevington et al., 2004). The physical domain is composed of the following subjects: pain and discomfort; energy and fatigue; sleep and rest; mobility; activities of daily living; dependence on medication or treatments to live; and capacity for working. The psychological domain is about: positive feelings; thinking, learning; memory; concentration; self-esteem; body image; appearance; negative feelings; and spirituality/religion/personal beliefs. The social relations domain has questions about personal relations, social support, and sexual activity. Lastly, there’s the environment domain that involves: physical security and protection; financial resources; availability and quality of social and health care; environment at home; opportunities to obtain new information and skills; participation and opportunities for leisure/recreation; physical environment aspects (pollution/noise/traffic/weather), and transport. The overall perception of QoL was based on the first question. This score rates 1 to 5. For the other domains, the mean was calculated, and subsequently, this value was multiplied by 4. After that, a score was obtained for each domain ranging from 4 to 20 (Skevington et al., 2004; Huang et al., 2006). A higher score indicates a better QoL (Khayyat et al., 2019).

### Possible Confounding Variables

The possible confounding variables of the relationship between PS and EF, coping, and QoL considered in this study were: age, sex, education, systolic blood pressure, and depression symptoms. Depression symptoms were evaluated by the Beck Depression Inventory (BDI) and were classified as follows: 0 to 11 points: minimal/no depression; 12 to 19 points: mild symptoms; 20 to 35 points: moderate symptoms; and 36 to 63 points: severe symptoms (Beck et al., 1996). The sociodemographic variables were acquired by self-reported questionnaires. The hemodynamic data (systolic and diastolic arterial blood pressure) was recorded beat to beat for 10 min using the Finometer^®^ (Finometer, FMS, Finapres Medical System, Holland). After that, the mean systolic and diastolic arterial blood pressure was obtained with the software program BeatScope, which used BP curves and patient information (age, sex, weight, and height) to calculate systolic and diastolic BP (Atala et al., 2015).

### Statistical Analyses

We described the characteristics of the sample using mean and SD or relative frequencies (n,%). The normality of quantitative variables was analyzed with the Shapiro–Wilk test. PS score was considered as the independent variable. The dependent variables were the composite Z-scores of the EF and the scores of the main coping responses and of all QoL domains. Each variable was considered separately in models adjusted for age, sex, education, systolic blood pressure, and depression symptoms. The association of the PS with EF performance, coping, and QoL was investigated using multiple linear regression models. Normal quantile-quantile (Q-Q) plots of the residuals and plots of the residuals versus the predicted values, as well as histograms of the residuals, were used to assess the assumptions of linear regression. The absence of multicollinearity was verified using the variance inflation factor. The statistical analyses were performed using the software R (v 4.0.0). The *p*-value was considered significant when ≤ 0.05.

## Results

Of the 48 participants who had complete data on the PS scale, we excluded one that presented an IQ less than 80, one that had a previous diagnosis of head trauma, and one with the diagnosis of epilepsy. Forty-five patients met the eligibility criteria and were included in the analysis of this cross-sectional study. The clinical and sociodemographic profile of the sample is described in Table 1. The mean age of the sample was 58.42 years (SD ± 8.9). Besides this, 32 of the subjects were female (71.11%) and the mean duration devoted to education was 12.13 years (SD ± 3.7). As for the clinical characteristics, the time of hypertension since diagnosis was 18.03 years (SD ± 11.07), and the systolic blood pressure (SBP) mean of the evaluated patients was 141.7 mmHg (SD ± 11.07), and the diastolic blood pressure (DBP) was 73.05 mmHg (SD ± 8.97). The mean number of medications in use was 4.37 (SD ± 1.95). As seen in Table 1, 17.77% of the sample presented moderate or severe depression symptoms, and the mean IQ was 96.4. The mean scores of the main coping responses and of all domains of the QoL are displayed in Supplementary Tables 1, 2, respectively. The mean scores of each EF test are also individually presented in Supplementary Table 3.

**TABLE 1:** Clinical and sociodemographic characteristics of the sample (*n* = 45).

		All (*n* = 45)
Age (years), mean (SD)		58.42 (8.9)
Sex, *n* (%)		
	Female	32 (71.11)
	Male	13 (28.88)
Race, *n* (%)		
	White	20 (44.44)
	Brown	15 (33.33)
	Yellow	2 (4.44)
	Black	8 (17.77)
Marital status, *n* (%)		
	Single	7 (15.55)
	Married	31 (68.88)
	Divorced	4 (8.88)
	Widower	3 (6.66)
Schooling (years), mean (SD)		12.13 (3.7)
Monthly income (USD*), mean (SD)		1108.94 (792.13)
Hypertension time since diagnosis (years), mean (SD)		18.03 (11.07)
Systolic Blood Pressure (mmHg), mean (SD)		141.7 (11.07)
Diastolic Blood Pressure (mmHg), mean (SD)		73.05 (8.97)
Cardiac Frequency (bpm), mean (SD)		66.10 (10.14)
BMI (kg/m^2^), mean (SD)		30.96 (6.05)
Number of drugs, mean (SD)		4.37 (1.95)
BDI score, mean (SD)		12.4 (7.60)
BDI > 19, *n* (%)		8 (17.77)
Intelligence Quotient, mean (SD)		96.4 (9.48)

*1 USD = 5.48 Real; SD = standard deviation; BMI = body mass index; BDI = Beck Depression Inventory.

### Perceived Stress and Executive Function

PS was not associated with EF in univariate (β−0.02; *p* = 0.11) and multiple linear regression models (β = −0.01; *p* = 0.20; β = < 0.01; *p* = 0.80) (Table 2).

**TABLE 2:** Association of perceived stress with executive function, coping strategies, and quality of life *n* = (45).

	Perceived Stress								
	Model 1			Model 2			Model 3		
	β	95% CI	*p*	β	95% CI	*p*	β	95% CI	*p*
Composite Z-score of EF	−0.02	−0.05 to 0.005	0.11	−0.01	−0.04 to 0.009	0.20	0.004	−0.02 to 0.03	0.80
Problem-focused	−0.06	−0.20 to 0.07	0.37	−0.07	−0.22 to 0.07	0.33	−0.05	−0.23 to 0.13	0.61
Emotion-focused	−0.20	−0.38 to −0.02	0.03	−0.25	−0.41 to −0.07	0.01	−0.23	−0.43 to −0.02	0.03
Dysfunctional coping	0.23	0.03 to 0.42	0.02	0.24	0.03 to 0.44	0.02	0.19	−0.05 to 0.42	0.13
Overall perception of QoL	−0.13	−0.22 to −0.04	< 0.01	−0.14	−0.22 to −0.05	< 0.01	−0.10	−0.20 to 0.002	0.06
Physical domain of QoL	−0.06	−0.15 to 0.03	0.18	−0.06	−0.16 to 0.03	0.20	−0.02	−0.12 to 0.09	0.77
Psychological domain of QoL	−0.19	−0.45 to 0.06	0.14	−0.20	−0.46 to 0.06	0.14	−0.03	−0.32 to 0.26	0.84
Social relations domain of QoL	−0.14	−0.24 to −0.03	0.01	−0.15	−0.26 to −0.04	0.01	−0.10	−0.24 to 0.03	0.14
Environment domain of QoL	−0.10	−0.18 to −0.02	0.01	−0.10	−0.11 to 0.01	0.03	−0.06	−0.16 to 0.04	0.25

*Linear regression models. EF = executive function; QoL = Quality of Life. Model 1: univariate model. Model 2: adjusted for age, sex, and education. Model 3: adjusted for age, sex, education, systolic blood pressure, and depression.*

### Perceived Stress and Quality of Life

Higher scores of PS were associated with a worse overall perception of QoL (β = −0.13; *p* < 0.01), worse QoL on social relations domain (β = −0.14; *p* = 0.01) and worse QoL on the environment domain (β = −0.10; *p* = 0.01) in univariate analyses (Table 2). However, in the adjusted models, PS was not an independent predictor for any QoL domain, although there was a tendency to predict the overall perception of QoL (β = −0.10; *p* = 0.06) (Table 2).

### Perceived Stress and Coping Strategies

In the univariate linear regression models, higher scores of PS were associated with a lower frequency use of emotion-focused strategies (β = −0.20; *p* = 0.03) and a greater use of dysfunctional coping (β = 0.23; *p* = 0.02). However, in the adjusted analyses, higher PS remained an independent predictor for a lower frequency of use of emotion-focused strategies only (β = −0.23; *p* = 0.03) (Table 2).

## Discussion

The purpose of this cross-sectional study, was to examine the association of PS with EF performance, coping strategies, and QoL in a group of hypertensive individuals. We found that higher scores of PS were related to lower scores of emotion-focused coping strategies. However, we did not find a significant association of PS with EF, QoL, and problem-focused and dysfunctional coping strategies.

These data are in agreement with other studies that used the same methods to measure PS and coping strategies in other populations. Saczuk et al. (2019) observed that the clinical group of adults diagnosed with clinical bruxism symptoms had a significantly higher percentage of individuals with high PS than the control group (28.6 vs. 4.0%). Besides this, the control group chose more often the acceptance and the religion as strategies to deal with stress in comparison with the study group. That is, positive coping strategies, mainly emotion-focused strategies, were chosen most frequently in the control group. Altunan et al. (2021) found similar results. In their study with individuals diagnosed with multiple sclerosis, patients with low PS levels used the acceptance strategy more than the ones with high PS.

Chronic high levels of distress may be indicative of poorly emotional regulation (Kubzansky et al., 2011). A study that assessed psychological measures such as social support, emotional regulation, and cognitive appraisal of the stressful situation showed that cortisol reactivity and norepinephrine secretion were highest in hypertensive men with poorer hedonistic emotional regulation (Wirtz et al., 2006).

During their lives, every people faces difficult and stressful situations capable to induce a variety of emotions. The emotions generate a disequilibrium that naturally demands an adaptation of the organism. This adaptation occurs through emotion regulation strategies. In this way, the balance is restored, and also the feeling of physical and psychological well-being. Therefore, emotion regulation can be characterized by these processes involved in the way of dealing with high levels of positive and negative emotions (McRae, 2016).

On the other hand, we did not find an association of PS with EF and QoL, although there was a tendency for the PS to predict the overall perception of QoL (β = −0.10; *p* = 0.06). Korten et al. (2017) also examined the association between PS (PSS-10) and EF (digit span and phonemic and semantic fluency), but in a bigger sample of 1,099 older adults. They observed a negative association between the general score of the PSS-10 and digit span backward in models adjusted for age, sex, education, function limitations, depression, and mastery (Korten et al., 2017). However, the authors did not include hypertension as a possible confounder variable, although it is a well-known risk factor for executive impairment (Iadecola et al., 2016). In this study, we found that the association between PS and EF was not independent of systolic blood pressure.

Evidence from longitudinal studies also showed associations between PS and cerebrovascular disease markers in an elderly cohort (Aggarwal et al., 2014). Aggarwal et al. (2014) found that each one-point increase in the PSS-4 score was associated with lower total brain volume and 7% greater odds of infarction after adjusting for history of hypertension and other covariables (Aggarwal et al., 2014). Another recent longitudinal research that evaluated the association of PSS-4 and executive functioning in old age showed that greater PS in the first wave of data collection predicted a steeper subsequent decline in executive functioning (Ihle et al., 2020). Moreover, the authors examined whether this longitudinal relationship differed by markers of cognitive reserve taking into account sociodemographic and clinical covariates. They verified the longitudinal relationship between PS and subsequent decline in executive functioning might be attenuated in individuals who have accumulated greater cognitive reserve through an engaged lifestyle (Ihle et al., 2020).

Regarding the association between PS and QoL, our results differ from others found in the literature. Meira et al. (2020) found a negative correlation between PS and all QoL domains: physical, psychological, social, and environmental in undergraduate students. They also found that higher PS was associated with less sleep duration and leisure time. According to the authors, these factors can negatively impact their social relationships and, consequently, worsen their health and QoL (Meira et al., 2020). Undergraduate students are exposed to different types of emotional stressors. In addition to this, they can deal differently with stressful situations. Factors that may help explain the difference between our findings.

Nevertheless, our results demonstrated that the expansion of the cognitive and behavioral repertoire to deal with stressful situations, with a focus on increasing the use of emotion-focused coping strategies, could be important psychological strategies for hypertensive patients with high levels of PS. In this sense, future research should focus on developing interventions for hypertensive patients to be fully assisted through programs aimed at emotional regulation, and adaptive coping strategies learning for the effective reduction of stress.

Finally, it is important to consider some of our study limitations. In the first place, this is a cross-sectional study design. In this way, we can not assume causality. Furthermore, the small sample size also could impact the effect size. This is an important issue that will be solved by larger further studies. Despite that, it was the first study that verified the relationship between PS and EF, coping strategies, and QoL in hypertensive patients, adjusting for sociodemographic and clinical control variables. Our results will be helpful for further investigations. Besides that, we included a variety of methods to assess EF that are extensively used in literature (Ferreira and Cunha, 2015; Moraes et al., 2019; Paradela et al., 2021), although they have low ecological validity. That is, they did not evaluate the emotional/motivational component of the EF. Furthermore, although we adjusted our analyses for some demographic and clinical variables, it is possible that unmeasured confounders were present, such as mental disorders or other clinical conditions.

## Conclusion

We found that higher scores of perceived stress were associated with lower scores of emotional-focused positive coping responses. However, perceived stress was not related to problem-focused and dysfunctional coping, executive function performance, or any domain of quality of life.

## Data Availability Statement

The raw data supporting the conclusions of this article will be made available by the authors, without undue reservation.

## Ethics Statement

The studies involving human participants were reviewed and approved by Comitê de ética do Hospital das Clínicas da Faculdade de Medicina da Universidade de São Paulo. The patients/participants provided their written informed consent to participate in this study.

## Author Contributions

LT, RP, and MI: conception and design of the work. LT, RP, and LM: acquisition, analysis, or interpretation of data for the work. DC, RP, and MI: drafting the work or revising it critically for important intellectual content. All authors contributed to the article and approved the submitted version.

## Conflict of Interest

The authors declare that the research was conducted in the absence of any commercial or financial relationships that could be construed as a potential conflict of interest.

## Publisher’s Note

All claims expressed in this article are solely those of the authors and do not necessarily represent those of their affiliated organizations, or those of the publisher, the editors and the reviewers. Any product that may be evaluated in this article, or claim that may be made by its manufacturer, is not guaranteed or endorsed by the publisher.
