# Supplementary material for: Higher Perceived Stress as an Independent Predictor for Lower Use of Emotion-Focused Coping Strategies in Hypertensive Individuals
Source: Front Psychol. 2022 May 24;13:872852. doi: 10.3389/fpsyg.2022.872852 (PMC9171365; doi:10.3389/fpsyg.2022.872852)
Supplement: Supplementary file 1 [file Table_1.docx]

**Supplementary Material**

Supplementary Table 1. The mean score of the 28-item Brief Coping Orientation for Problem Experienced (Brief COPE) questionnaire organized according to the main strategies (*n* = 45)

|  | All (n = 45) | Min | Max |
| --- | --- | --- | --- |
| Problem-focused, mean (SD) | 17.13 (3.30) | 9 | 23 |
| Emotion-focused, mean (SD) | 26.69 (4.38) | 15 | 36 |
| Dysfunctional coping, mean (SD) | 25.87 (4.61) | 18 | 39 |

SD = standard deviation.

Supplementary Table 2. The mean score of all domains of the World Health Organization Quality of Life Questionnaire (WHOQOL-bref) (*n* = 45)

|  |  | All (n = 45) |
| --- | --- | --- |
| Overall perception of QoL |  | 14.27 (2.24) |
| Physical, mean (SD) |  | 13.65 (2.18) |
| Psychological, mean (SD) |  | 18.14 (6.03) |
| Social relations, mean (SD) |  | 14.25 (2.64) |
| Environment, mean (SD) |  | 13.81 (2.01) |

QoL = Quality of Life; SD = standard deviation

Supplementary Table 3. The mean scores of each executive function test (*n* = 45)

|  |  | ***n = 45*** |
| --- | --- | --- |
| Global executive function, mean (SD) |  | 0.01 (0.64) |
| FAB (points), mean (SD) |  | 14.33 (2.29) |
| FAS (points), mean (SD) |  | 29.89 (9.91) |
| LNS (points), mean (SD) |  | 6.95 (2.77) |
| Digit span forward (points), mean (SD) |  | 5.86 (2.2) |
| Digit span backward (points), mean (SD) |  | 4.34 (1.58) |
| WCST, mean (SD) |  | 1.82 (1.13) |
